# Growth, Hematobiochemical Changes, and Gut Bactericidal Potential of Atama, *Heinsia crinita,* Leaf Extract–Based Diet on Nile Tilapia

**DOI:** 10.1155/sci5/5594984

**Published:** 2025-06-29

**Authors:** Umma Samuel B., Ibrahim Adeshina, Samuel Sabo, Sajjad Pourmozaffar

**Affiliations:** ^1^Department of Fisheries and Aquaculture, Federal University Wukari, Wukari, Taraba, Nigeria; ^2^Department of Fisheries and Aquaculture, University of Ilorin, Ilorin, Nigeria; ^3^Persian Gulf Mollusks Research Station, Persian Gulf and Oman Sea Ecology Research Center, Iranian Fisheries Sciences Research Institute (IFSRI), Agricultural Research Education and Extension Organization (AREEO), Bandar-e-Lengeh, Iran

**Keywords:** Atama leaf extract, bactericidal potential, growth, medicinal value, Nile tilapia

## Abstract

Feed additives are essential for economic sustainability in aquaculture. However, the use of synthetic chemical additives in fish culture can lead to antibiotic resistance, reduced drug potency, and decreased profitability. Hence, the need for an eco-friendly alternative such as Atama, *Heinsia crinita*, leaf. Therefore, the presence of metabolites was characterized in Atama leaves extract (ALE) using a gas chromatography–mass spectrophotometer (GC–MS), while minimum inhibitory concentration (MIC) and zone of inhibition (ZI) were determined against some selected pathogenic organisms using standard methods. Furthermore, the effects of dietary ALE on growth performance, intestinal microbiota, hematology, and liver function enzymes were examined in Nile tilapia, *Oreochromis niloticus*. Four diets of 350 g/kg crude protein were formulated and fortified with ALE at 0.0 (control), 2.0, 4.0, or 6.0 g/kg diet. Each diet was fed to Nile tilapia juveniles (4.0 ± 0.63 g) to apparent satiation three times a day for 56 days. The GC–MS analysis of ALE revealed nine metabolites, with methyl stearate being the most abundant (29.1%) and neophytadiene the least (0.4%). ALE significantly (*p* < 0.05) inhibited the all-tested organisms with MIC 2.0 mg/mL and ZI ranged from 12.6 to 30.2 mm. Also, it was observed that ALE–enriched diets linearly significantly (*p* < 0.05) promoted fish growth, feed intake, and specific growth rate than the fish fed control diet. Conversely, the feed conversion ratio was greatly reduced (*p* = 0.001) in fish fed ALE–based diet than the basal group without major effect on the survival rate. Total viable counts of bacteria were linearly and quadratically significantly (*p* < 0.05) decreased in Nile tilapia fed with ALE–enriched diets when compared to the fish fed the control diet in an inverse relationship with ALE inclusion levels. The population of *Vibrio* Sp. (24.2 × 10^6^ CFU/g), *Flavobacterium* sp. (3.7 × 10^6^ CFU/g), *Proteus* sp. (1.6 × 10^6^ CFU/g), and *Pseudomonas* sp. (52.0 × 10^6^ CFU/g) in the control group were greatly reduced in fish fed 4.0 g ALE/kg diet to 7.7 × 10^6^ CFU/g, 2.3 × 10^6^ CFU/g, 1.1 × 10^6^ CFU/g, and 1.3 × 10^6^ CFU/g, respectively. Hematological profiles and liver function indices significantly decreased at 4–6 g ALE/kg diet. In conclusion, ALE–supplemented diets inhibited the growth of selected pathogenic bacteria, promoted fish growth, improved hematological profiles, and reduced liver function enzyme activity in Nile tilapia when fed diets containing 4.0–6.0 g/kg ALE.

## 1. Introduction

Aquaculture production has increased steadily and has become the fastest growing food production sector in recent years, significantly contributing to global fish supply. This growth aims to meet the rising demand for fish and fish products while also serving as a key component of development and poverty reduction strategies worldwide [1]. Approximately 40% of aquaculture-produced fish comes from tilapia farming [2]. The Nile tilapia, *Oreochromis niloticus* and tilapia farming generally, is considered as one of the most important species of fish in tropical and subtropical aquaculture and also a model of high tolerance to a wide range of environmental conditions contributing to enhancing food security and reducing malnutrition [3–6]. To improve its production, feed additives which are substances needed by the body in minute quantities are now introduced into animal feeds to provide such benefits as boosting fish growth and optimizing the prime health of fish [7]. The excessive use of chemicals and antibiotics in fish culture has led to issues such as antibiotic resistance, reduced drug efficacy, and declining profitability [8]; hence, the need for an eco-friendly alternative.

The use of different types of feed additives in aquaculture is now on the increase as growth promoters and as boosters for feed intake, digestion, nutrient bioavailability, and immunity [9–15]. Herbal products, in particular, have been recognized for their antistress, appetizing, tonic, and antimicrobial properties, offering protection against several diseases [16–20]. For example, Atama, *Heinsia crinita,* widely grown in Nigeria and many African countries has medicinal values [21, 22] with high levels of alkaloids, flavonoids, saponins, tannins, cardiac glycosides, terpenes, anthranoids, and anthraquinones [23, 24]. Despite the potential of Atama as a growth stimulant and gut microbiota modulator, research on its inclusion in fish diets remains limited. Hence, for a successful tilapia culture, a nutritionally balanced diet with a low cost of production is important. Therefore, this study investigated the growth and the hematobiochemical changes of *O. niloticus* fed Atama leaves extract (ALE)–based diets.

## 2. Materials and Methods

### 2.1. Preparation of Extract and Metabolites Analysis

Fresh Atama, *H. crinita,* leaves (1.2 kg) were purchased from the market, rinsed with clean water, and soaked in 3.8 L of ethyl acetate (INEOS, Grangemouth, FK3 9XB, United Kingdom) for 72 h to extract the phytochemicals to produce ALE [25]. Ethyl acetate was chosen due to its balanced polarity, volatility, nonhazardous nature, and inertness, making it suitable for extracting organic compounds. In addition, it is easily removable by evaporation, poses minimal environmental hazards, and does not readily undergo side reactions [25]. Afterward, the ALE was exposed to a fumes cupboard to vaporize the solvent and recover the extracted solute.

ALE was examined using gas chromatography–mass spectrometry (GC–MS). In brief, 1 μL of ALE was injected in Agilent 7890A gas chromatography with Agilent 5975 mass spectrometer detector (Avondale, PA, USA) equipped with an HP-5ms capillary column (5 m length, 0.25 mm diameter, and 0.25 μm internal diameter). The oven temperature was initially set at 35°C and held for 5 min, and then increased at a rate of 20°C per minute to 250°C, when it was held for 5 min. Helium was used as the carrier gas at a flow rate of 1 mL per minute. The samples were run using full scan with a range of 50–750 mass units and recorded using an HP ChemStation System. The extracted compounds were identified by comparing their relative retention times (RTs) and mass spectra with authentic reference standards from a database [25, 26]. The NIST XI library database was used, selecting only compounds with a 90% or higher probability for structural assignments. The two metabolites with the highest concentrations were selected for susceptibility testing.

#### 2.1.1. Bacteria Inhibition Assay

Sterile water (10 mL) was dispensed into nine test tubes (equal to a number of isolates to be tested, i.e., *Escherichia coli, Salmonella* sp.*, Shigella* sp.*, Vibrio* sp.*, Bacillus* sp.*, Enterobacteriaceae* sp.*, Flavobacterium* sp.*, Proteus* sp., and*, Pseudomonas* sp.). The bacterial broth cultures were serially diluted to a 1:100 dilution factor using sterile water [27]. From the diluted broths, 0.1 mL of each isolate broth (1 × 10^7^ CFU/mL) [26] was added to each tube and used immediately [28].

#### 2.1.2. Concentration and Application of the Extracts

ALE was dissolved in dimethyl sulfoxide (DMSO) to produce a two-fold dilution to constitute 0, 1, 2, 3, and 4 mg/mL using agar well diffusion methods [25, 26, 29, 30]. A 6 mm cork borer was used to create wells in nutrient agar plates. Each well was filled with the ALE using sterilized droppers and incubated at 37°C for 24–48 h [31] for each bacterial isolate. Sterile disposable Petri dishes were swabbed with 0.1 mL of fresh 24 h culture of each isolate using swab sticks, in triplicate.

#### 2.1.3. Determination of Minimal Inhibitory Concentration (MIC) and Zone of Inhibition (ZI) Assay

MICs were determined for each bacterial isolate as described by the National Committee for Clinical Laboratory Standards [28]. In brief, the microdilution method was used, where ALE was serially diluted (two-fold). The MICs were determined using the agar well diffusion method, and inoculated plates were incubated at 37°C for 18 h. The MICs were determined as the lowest concentration of the extracts inhibiting the visible growth of each organism on the agar plates. The ZIs were measured with a meter ruler calibrated in millimeters.

### 2.2. Diet Preparation and Culture of Nile Tilapia

Pulverized fish-feed ingredients were used to prepare 35% crude protein isonitrogenous diets fortified with ALE at 0.0, 2.0, 4.0, or 6.0 g/kg (Table 1). Ingredients were thoroughly mixed with warm water (100 mL per 1000 g dry matter [DM]) to form dough and were pelleted using a 2 mm pellet-size dry pelleting machine (Model: LM50, Henan Lima Machinery Manufacture Co. LTD., Zhengzhou, China). The pelleted feeds were produced in batches of 1 kg fortnightly and stored at 4°C in a refrigerator to maintain its freshness [32]. The proximate composition was carried out as described by the standard methods of the Association of Official Analytical Chemists [33].

The Nile tilapia, *O. niloticus*, fingerlings were obtained from a reputable fish farm in the Ibi local government area of Taraba State, Nigeria, and acclimatized for 14 days during which the fish were fed with a commercial feed (Aller Aqua, Allervej 130, 6070, Christiansfeld, Denmark). After acclimatization, fish (mean weight = 4.0 ± 0.63 g) were randomly assigned to four experimental tanks (50 L each) in a completely randomized design, with three replicates per treatment and fed one of the experimental diets up to satiation three times daily for 56 days as described by Adeshina et al. [34]. The temperature, dissolved oxygen (DO), and pH of the culture water were monitored daily using a mercury-in-glass thermometer, digital DO meter (Model AVI-660, Labtech International Ltd., Heathfield, UK), and digital pH meter (Model: Photoic 20, Labtech International, Ltd). Their values were within the recommended levels ranging from 28.5 to 28.0°C, 6.38 to 6.07 mg/L, and 6.93 to 6.83, respectively.

Growth data were taken after the study period and data on growth were calculated using the following equations:

(1) $$\begin{array}{c} \text{weight gain }\left(\text{WG},g\right)=\text{final body weight}\left(g\right)-\text{initial body weight}\left(g\right), \end{array}$$

(2) $$\begin{array}{c} \text{specific growth rate }\left(\text{SGR},\%g/\text{day}\right)=\frac{\log\;eW_{2}-\log\;eW_{1}}{\text{duration of culture}\left(\text{days}\right)}\times 100, \end{array}$$

(3) $$\begin{array}{c} \text{feed conversion ratio }\left(\text{FCR}\right)=\frac{\text{fish feed intake}\left(g\right)}{\text{fish weight gain }\left(g\right)}, \end{array}$$

(4) $$\begin{array}{c} \text{survival rate }\left(\text{SR},\%\right)=\frac{\text{number of fish sto}c\text{ked}-\text{mortality}}{\text{stocking no}.\text{ of fish stocked}}\times 100. \end{array}$$

### 2.3. Hematological Assay

At the end of the 56 days study, the experimental fish were fasted for 24 h and sedated as described by Adeshina et al. [35], using 30 mL of tricaine methanesulfonate buffered with sodium bicarbonate (MS222, Syndel, Ferndale, Washington, USA) for 5 min. Blood samples were randomly collected from fish in each tank using a 1 mL syringe (BD Safety-Lok Syringe and Needle, Becton Dickinson, New Jersey, US). Blood was dispensed into two different sampling bottles: The first contained ethylenediaminetetraacetic acid (EDTA) as an anticoagulant for hematological analysis as described by Mitruka and Rawnsley [36]. In brief, packed cell volume (PCV, %), red blood cells (RBCs, × 10^6^/μL), white blood cells (WBCs, × 10^3^/μL), and platelets (PLTs, × 10^6^/μL) [37, 38], while hemoglobin (Hb, g/dL) was determined by using Vankampen and Ziglstra's method [39]. The Wright–Giemsa stain method was used to estimate differential counts of lymphocytes (Lym, %), heterocytes (Het, %), monocytes (Mon, %), eosinophils (Eos, %), and basophils (Bas, %) using a light microscope (Olympus CX21, Japan, 1000x magnification).

### 2.4. Biochemical Assay

The second sampling bottle, from Section 2.3, was without anticoagulant for serum biochemical analysis as described by Schmidt and Schmidt [40]. According to the Reitman and Frankel [41] method, liver function enzymes (aspartate aminotransferase [AST, IU/L], alanine aminotransferase [ALT, IU/L], and alkaline phosphatase [ALP, IU/L]) were measured colorimetrically [42], while the creatinine concentrations were determined using the techniques developed by Coulombe and Favreau [43].

### 2.5. Evaluation of Intestinal Microbiota of Nile Tilapia Fed ALE–Based Diets

At the end of the feeding period, the intestinal tracts of the cultured fish were aseptically removed for microbial analysis. The selective agars for the identification of the gut flora were prepared according to the manufacturer's instructions (Oxoid Ltd, United Kingdom). In brief, *Pseudomonas* Centrimide Agar was prepared for *Pseudomonas* sp., violet red bile glucose agar (VRBGA) was prepared for *Enterobacteriaceae species, Bacillus* selective agar base was prepared to isolate *Bacillus species,* eosin methyl blue (EMB) agar was prepared for the identification of *Escherichia coli, Proteus* triple sugar iron (TSI) was prepared for *Proteus* species, *Vibrio* Agar (M820) was prepared for isolation of *Vibrio* species, *Salmonella* and *Shigella Agar* (SSA) was prepared for the identification of *Salmonella* and *Shigella* species, and tryptic soy agar (TSA) was prepared to isolate *Flavobacterium* species, respectively [44].

### 2.6. Statistical Analysis

The Anderson–Darling normality test was performed to assess the normality of data distribution and homogeneity of variance. In addition, Grubb's test was conducted to detect potential outliers in the dataset. Data obtained from the growth performance, hematobiochemical analysis, and gut microbiota activity were subjected to one-way analysis of variance (ANOVA). Tukey's pairwise comparison test was used as the follow-up to separate the means that were significantly different. The optimum inclusion level for dose-response relationships was analyzed using orthogonal contrasts (linear and quadratic) at a 5% probability level. The statistical analysis was carried out on the Minitab statistical software program Version 19 (Minitab LLC, Pennsylvania, USA).

## 3. Results

### 3.1. Distribution and Quality of Identified Metabolites in ALE

**Figure 1:**
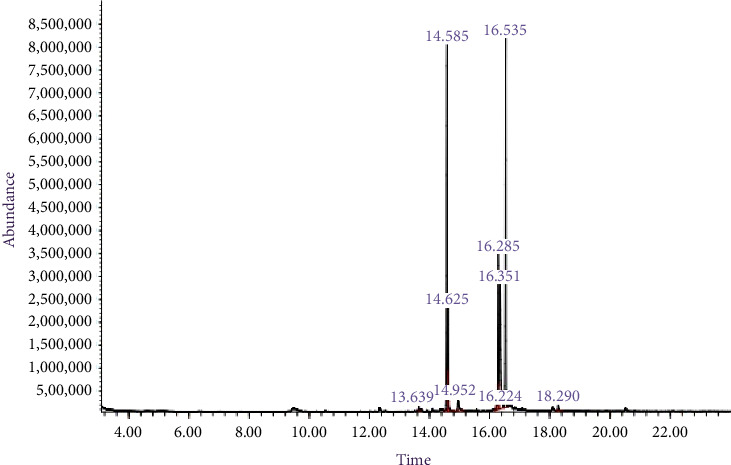
Chromatogram of Atama leaves extract showing the detector response (abundance) against the retention time.

The metabolites identified using GC–MS indicated nine compounds at varied RTs in the 1.57 g ALE sample subjected to analysis. Methyl stearate was the most abundant with 29.1% area cover out of 100.0% distribution, followed by pentadecanoic acid, 14-methyl- with 28.5%, while the least abundant was neophytadiene with 0.4% area cover (Figure 1). On a quality scale of 0–100, methyl stearate, 9-octadecenoic acid (Z)-, methyl ester, and 9-octadecenoic acid, methyl ester (E) had the highest quality at 99, while neophytadiene maintained the least quality at 58.

### 3.2. MIC and ZI of ALE Against Tested Organisms

Table 2 shows the MIC of the ALE against the nine tested isolates. ALE of 2.0 and above showed inhibition of the isolates, while the control and 1.0 ALE mg/mL showed little or no inhibition against the isolates. Thus, the result showed that the MIC is 2.0 mg ALE/mL (Table 2). Significant differences (*p* < 0.05) were observed in ZIs observed in isolates exposed to different ALE levels (Table 2). All isolates exhibited significantly higher (*p* < 0.05) sensitivity to ALE when compared to the control especially from 2.0 to 4.0 mg/mL revealing a ZI ranging from 12.6 to 30.2 mm (Table 3).

### 3.3. Growth Performance of Nile Tilapia Fed ALE–Based Diets

**Figure 2:**
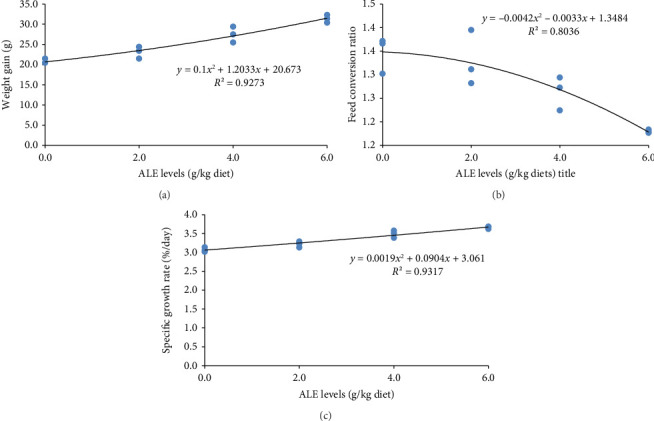
The relationship between weight gain (g), feed conversion ratio, and specific growth rate and ALE levels.

The ALE levels in this study linearly but not quadratically enhanced the growth performance of Nile tilapia (*p* ≤ 0.001). This indicates significantly higher final body weight (g), WG (g), feed intake (g), and SGR (%/day) than their counterpart fed the control diet with their corresponding higher values obtained in fish fed 6.0 g ALE/kg diet (Table 4). Conversely, the FCR was greatly reduced (*p* < 0.001) in fish fed ALE–based diet than the basal group (Table 4). However, the SR was not linearly and quadratically affected in Nile tilapia fed ALE dietary diets (Table 4). The relationships between WG (g), FCR, and SGR (%/day) and ALE levels (Figure 2) were best expressed by the second-order polynomial regressions as *y* = 0.1*x*^2^ + 1.2033*x* + 20.673 (*R*^2^ = 0.9273), *y* = −0.0042*x*^2^ − 0.0033*x* + 1.3484 (*R*^2^ = 0.8036), and *y* = 0.0019*x*^2^ + 0.0904*x* + 3.061 (*R*^2^ = 0.9317), respectively. This relationship evoked that the optimum ALE level for Nile tilapia is found to be a 6 g/kg diet.

### 3.4. Intestinal Microbiota of Nile Tilapia Fed ALE–Based Diets

Total viable counts of bacteria, pathogenic, and opportunistic bacteria were found to be linearly and quadratically significantly (*p* < 0.05) decreased in Nile tilapia fed with ALE–enriched diets when compared to the fish fed the control diet (Table 5). Furthermore, the reductions were inversely proportional to ALE inclusion levels (Table 5). In the same order, the population of pathogenic and opportunistic bacteria, especially, *Vibrio sp., Flavobacterium* sp.*, Proteus* sp., and *Pseudomonas* sp., was greatly reduced in fish fed 4.0 g ALE/kg diet from 52.0 × 10^6^ CFU/g in *Pseudomonas* sp. to 1.1 × 10^6^ CFU/g in *Proteus* sp. (Table 5).

### 3.5. Hematological Profiles of Nile Tilapia Fed Dietary ALE Levels

Significant elevations in PVC, Hb, RBC, WBC, PLT count, and lymphocytes were noticed to be increased linearly and quadratically in fish fed on graded ALE levels in a dose-dependent manner where their highest values were observed with fish fed 6 g ALE/kg diet (*p* < 0.05; Table 6). In a dose-dependent form, significant linear and quadratic decreases in heterocytes, monocytes, eosinophils, and basophils percentages were noticed and their lowest values were observed in fish fed 4–6 g ALE/kg diet; whereas their highest values were obtained with fish fed with the control diet (*p* < 0.05; Table 6).

### 3.6. Liver Function and Kidney Indices of Nile Tilapia Fed ALE–Enriched Diets

**Figure 3:**
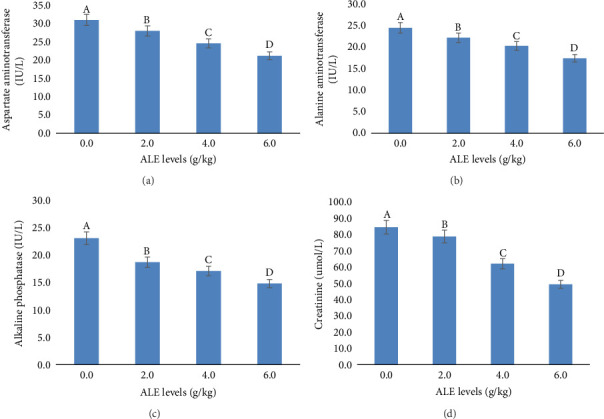
Liver functions indices of Nile tilapia fed ALE–based diets. Bars having the same superscript letter are not significantly different at *p* < 0.05. Note: ALE = Atama leaves extract.

In addition, the liver function indices AST, ALT, ALP, and creatinine were decreased significantly (*p* < 0.05) with the increase in ALE levels in fish diets especially at levels of 4–6 g/kg diet over that fed the control diet (Figure 3). The highest values of AST, ALT, ALP, and creatinine were recorded in fish fed control diet, while the least values were obtained in fish fed with 6.0 g ALE/kg diet, respectively (Figure 3).

## 4. Discussion

Analysis of metabolites using GC–MS has been used to profile the active compounds in plant extracts [25, 26, 45–48]. In this study, GC–MS profiling of the ALE has unveiled a spectrum of metabolites with potential bioactive properties. The presence of methyl stearate and other fatty acid esters in the ALE is important because these compounds have been associated with antimicrobial and anti-inflammatory activities [47, 48], thus underscoring the plant's medicinal potential. The detection of neophytadiene, albeit in low abundance, is also significant due to its reported antioxidant properties. The quality scores assigned to the identified compounds may reflect the confidence in their identification and potential purity. High-quality scores for methyl stearate and related compounds suggest a reliable identification.

The antimicrobial efficacy of the ALE was evaluated against nine bacterial isolates, with the MIC determined to be 2.0 mg/mL. At concentrations of 2.0 mg/mL and higher, ALE effectively inhibited the growth of all tested isolates showing ZI between 12.6 and 30.2 mm underscoring its antimicrobial activity. These findings are consistent with recent studies investigating the antimicrobial properties of *Ocimum gratissimum* and *Eugenia caryophyllata* extracts [25]. Their results demonstrated that ethyl acetate extract of these herbs exhibited ZI ranging from 11.7 to 17.0 mm against Gram-positive bacteria. Similarly, the extract of *Artemisia vestita* leaves showed notable antimicrobial effects, with inhibition zones up to 17.6 mm against *Escherichia coli* [49]. Comparatively, the MIC of ALE at 2.0 mg/mL is higher than the MICs reported by Konappa, et al. [48]. This discrepancy could be attributed to differences in phytochemical compositions, extraction methods, or the specific bacterial strains tested.

Nile tilapia showed significant linear growth improvement with ALE supplementation up to 6.0 g/kg. In addition, the reduced FCR in fish fed ALE–based diets indicates improved feed utilization efficiency without posing any major detrimental effects on fish health, especially its SR. Herbs and herbal products added to fish feed are reported to significantly promote growth, cure many diseases, reduce stress, improve immunity, and prevent infections in fish under culture and are as well eco-friendly [50, 51]. The findings of this study are in concomitant with the findings of Adeshina et al. [34] and Adeshina et al. [35], who reported significantly improved growth performance of Nile tilapia fed diets supplemented with the leaves' extracts of *Mitracarpus scaber* and *Tridax procumbens* at the rate between 4 and 6 g/kg. In addition, the growth performance of Nile tilapia was greatly enhanced when fed on 5.0 g of *M. oleifera* leaf extract/kg than the control group [52]. While the findings of the current study align with previous research supporting the efficacy of plant-derived additives in improving tilapia growth performance, key differences exist. Unlike the quadratic effects observed in some studies, the present research indicates a strictly linear enhancement with ALE supplementation. This suggests that ALE maintains its positive effects without evident diminishing returns within the tested range. This may be attributed to the efficacy of ALE additives in the Nile tilapia diet and also underscores the unique response patterns elicited by different metabolite compounds in ALE.

Feeding Nile tilapia with a diet enriched with ALE leads to a significant linear and quadratic reduction in total viable counts of bacteria, including pathogenic and opportunistic species, compared to a control diet. Notably, higher levels of ALE inclusion corresponded to greater reductions in bacterial populations, with specific decreases observed in pathogens such as *Vibrio* sp., *Flavobacterium* sp., *Proteus* sp., and *Pseudomonas* sp. As reported by Parveen et al. [53], Nile tilapia fed *M. oleifera–*based diets showed a lower presence of intestinal pathogenic bacteria, including *E. coli*, *P. aeruginosa*, *Shigella* sp., and *Salmonella* sp. This reduction in pathogenic bacteria further corroborated with the higher growth performance recorded in fish fed the fortified diets because the reduction in pathogenic bacteria stimulates the growth of beneficial bacteria which will enhance digestion and nutrient absorption and consequently improve growth. Once pathogenic gut bacteria could not colonize the fish gut, those that are nonpathogenic such as the autochthonous are able to colonize the mucosal surface, thereby playing important roles in producing extracellular enzymes and blocking the attachment site for pathogens and secreting a wide range of bacteriocin and promoting the bioavailability of nutrients and also other metabolic benefits in fish [54–56]. This study further reiterates the potential of dietary ALE as a prebiotics agent to modulate the gut microbiota of Nile tilapia, reducing pathogenic bacteria and enhancing beneficial microbial populations. In contrast, Elumalai et al. [57] found that dietary supplementation with *Bacillus amyloliquefaciens* nanoparticles increased the total bacterial count in the gut but enhanced the presence of beneficial bacteria. This shift in microbial composition could be associated with improved intestinal histomorphology of the fish and bioavailability of the active compounds in the additives.

Hematological examination is a commonly used method for fish physiological and health status assessment [58, 59]. The hematological parameters indicated an improved health status of the Nile tilapia fed ALE–based diet. This study revealed that dietary ALE on Nile tilapia significantly enhanced in a dose-dependent pattern, increases in PCV, Hb, RBC, and WBC, PLT, and lymphocytes, with the highest values observed at 6 g ALE/kg diet. Similarly, hematological profiles of Nile tilapia were evoked due to the fortification of the diets with *T. procumbens* [34], *M. scaber* [35], and *M. oleifera* [53] leaves extract. Phytochemicals assist in boosting the general health status and immunity of the fish [60]. In another study, Shirbot, *Barbus grypus*, demonstrated improved hematological indices when fed 5.0 g/kg of dietary *Aloe vera* extract. The study attributed these effects to the bioactive compounds in ALE, which may stimulate leukopoiesis and enhance immune responses. Monocytes are considered as the largest blood cell in fish and have the same functions as those of mammals including phagocytosis, antigen presentation, and the production of cytokines [61]. Basophils multiply in the bone marrow in response to inflammatory signals and are mobilized to the blood, spleen, lung, and liver [62]. They play the role of innate immune regulatory cells in allergy and immunotherapy [63]. Eosinophil according to Balla et al. [64] constitutes a distinct lineage of granulocytes that provide innate immune surveillance, assistance with T lymphocyte-mediated humoral immune response, and tissue remodeling through the release of granule components. Thus, it was observed that ALE influenced the build-up of some hematological parameters and also improved the immune factors of the ALE–fed Nile tilapia. Conversely, percentages of heterocytes, monocytes, eosinophils, and basophils decreased linearly and quadratically, reaching their lowest levels in fish fed a 4–6 g ALE/kg diet, compared to the control group. These observed decreases in the current study may indicate a shift towards a more efficient immune response, potentially reducing unnecessary inflammatory reactions.

Monitoring liver function enzymes in the blood is known to be a sensitive indicator of liver dysfunction and an essential method for determining the health status of fish, and in turn a good bioindicator of both the ingested food and environmental water quality [65]. These blood enzymes are part of the inactive enzymes predominantly found in fish liver tissue cells. The serum biochemical parameters included in this current study were seen to dwindle along the treated diets, with the least in the 6.0 g ALE/kg diet, an indication of the extract's positive effect on the fish. This trend of results was also reported by Adeshina et al. [34], where the AST, ALT, and ALP were seen to dwindle with the addition of dietary *Tridax procumbens* leaves extract, which was suggested to improve the well-being of the fish. The observed significant reductions in AST, ALT, ALP, and creatinine in fish fed diets supplemented with 4–6 g/kg of ALE suggest a hepatoprotective effect of ALE at these concentrations. This is evidenced by the highest enzyme levels in the control group and the lowest in the 6.0 g ALE/kg diet group which are in agreement with the authors in [34, 35, 52, 53], who reported a huge decline in fish fed plant extract–based diets.

## 5. Conclusion

This study demonstrated that ALE contains diverse bioactive metabolites, with methyl stearate as the most abundant and neophytadiene the least. ALE exhibited strong antimicrobial activity (MIC: 2.0 mg/mL) and dose-dependent inhibition. Incorporating ALE into Nile tilapia diets improved growth, with 6.0 g/kg yielding the best performance. It reduced pathogenic bacteria, enhanced hematological parameters, and improved immune cells. ALE also lowered liver and kidney indices, suggesting hepatoprotective and nephroprotective effects at 4.0–6.0 g/kg. These findings highlight ALE's potential as a functional dietary additive for enhancing growth, immunity, and organ health in Nile tilapia. Future research should explore its long-term effects and examine its histological alteration in the gut for sustainable fish production.

## Figures and Tables

**Table 1:** Gross and proximate composition of experimental diets' ALE levels (g/kg, dry matter).

Ingredients	ALE levels (g/kg)^1^			
	0.0	2.0	4.0	6.0
Fish meal (60% CP)	250.0	250.0	250.0	250.0
Soybean meal (44% CP)	400.0	400.0	400.0	400.0
Yellow maize (8% CP)	290.0	288.0	286.0	284.0
Starch	20.0	20.0	20.0	20.0
Vegetable oil	10.0	10.0	10.0	10.0
Vitamin premixes^2^	10.0	10.0	10.0	10.0
Mineral premixes^3^	20.0	20.0	20.0	20.0
ALE^1^	0.0	2.0	4.0	6.0
Crude protein	351	352	354	355
Crude fat	62	64	65	62
Crude fiber	45	43	44	44
Ash	89	95	90	91
Carbohydrate	321	310	320	320
Moisture content	90	90	90	90

^1^ALE = Atama leaves extract.

^2^Vitamin premix: vitamin A: 22;000 I.U; vitamin D3: 5000 I.U; vitamin E: 300 mg; vitamin K3: 10 mg; vitamin B1: 20 mg; vitamin B2: 25 mg; vitamin C: 300 mg; niacin: 120 mg; vitamin B6: 10 mg; vitamin B12: 0.05 mg; folic acid: 5 mg; biotin: 1 mg.

^3^Mineral premix: calcium pantothenate: 60 mg; choline chloride: 500 mg; inositol: 50 mg; manganese: 30 mg; iron: 35 mg; zinc: 45 mg; copper: 3 mg; iodine: 5 mg; cobalt: 2 mg; selenium: 0.15 mg; antioxidant: 80 mg.

**Table 2:** Minimum inhibitory concentration of ALE against the tested isolates.

Isolates	ALE concentrations (mg/mL)^1^				
	0.0	1.0	2.0	3.0	4.0
*Escherichia coli*	—	—	++	++	++
*Salmonella sp.*	—	—	++	++	++
*Shigella sp.*	—	+	++	++	++
*Vibrio Sp.*	—	—	++	++	++
*Bacillus sp.*	—	+	++	++	++
*Enterobacteriaceae sp.*	—	—	++	++	++
*Flavobacterium sp.*	—	—	++	++	++
*Proteus sp.*	—	—	++	++	++
*Pseudomonas sp.*	—	+	++	++	++

*Note:* — = No activity; + = inhibition less than 10 mm; ++ = inhibition (more than 12 mm diameter).

^1^ALE = Atama leaves extract.

**Table 3:** Zone of inhibitions (ZIs) of ALE against the tested isolates.

Isolates	ALE concentrations (mg/mL)^1^					Pooled StDev^2^	*p* value	
	0.0	1.0	2.0	3.0	4.0		Linear	Quadratic
	Zones of inhibitions (mm)							
*Escherichia coli*	0.0^b^	0.0^b^	18.3^a^	18.4^a^	18.9^a^	0.221	≤ 0.001	≤ 0.001
*Salmonella sp.*	0.0^b^	0.0^b^	22.1^a^	22.8^a^	23.0^a^	0.112	≤ 0.001	< 0.001
*Shigella sp.*	0.0^b^	0.5^b^	24.4^a^	24.5^a^	25.1^a^	0.356	≤ 0.002	≤ 0.001
*Vibrio Sp.*	0.0^b^	0.0^b^	15.4^a^	16.2^a^	16.5^a^	0.120	≤ 0.001	< 0.001
*Bacillus sp.*	0.0^c^	0.4^c^	26.3^b^	26.9^b^	30.2^a^	0.264	≤ 0.003	≤ 0.001
*Enterobacteriaceae sp.*	0.0^b^	0.0^b^	12.6^a^	12.9^a^	13.5^a^	0.215	≤ 0.001	≤ 0.001
*Flavobacterium sp.*	0.0^b^	0.0^b^	18.3^a^	19.4^a^	21.5^a^	0.114	< 0.001	≤ 0.001
*Proteus sp.*	0.0^d^	0.0^d^	20.3^c^	23.1^b^	25.2^a^	0.422	≤ 0.002	≤ 0.001
*Pseudomonas sp.*	0.0^d^	0.8^c^	19.4^b^	20.3^b^	22.4^a^	0.219	≤ 0.003	≤ 0.001

*Note:* Means having the different letters in the same row are significantly different at *p* < 0.05.

^1^ALE = Atama leaves extract.

^2^Pooled StDev = Pooled standard deviation.

**Table 4:** Growth performance of Nile tilapia fed ALE–based diets.

Parameters	ALE concentrations (g/kg)^1^				Pooled StDev^2^	*p* values	
	0.0	2.0	4.0	6.0		Linear	Quadratic
Initial body weight (g)	4.5^a^	4.6^a^	4.5^a^	4.6^a^	0.058	0.111	0.347
Final body weight (g)	25.3^c^	27.7^c^	32.0^b^	36.0^a^	1.384	≤ 0.001	0.328
Weight gain (g)	20.8^c^	23.1^c^	27.5^b^	31.4^a^	1.346	≤ 0.001	0.333
Feed intake (g)	28.0^c^	30.7^c^	34.7^b^	37.0^a^	0.145	≤ 0.001	0.213
Feed conversion ratio	1.4^a^	1.3^ab^	1.3^ab^	1.2^b^	0.040	≤ 0.001	0.181
Specific growth rate (%/day)	3.1^b^	3.2^b^	3.5^a^	3.7^a^	0.069	≤ 0.001	0.711
Survival rate (%)	95.6^a^	97.8^a^	95.6^a^	97.8^a^	3.849	0.667	1.000

*Note:* Means having the same superscript letter in the same row are not significantly different at *p* < 0.05.

^1^ALE = Atama leaves extract.

^2^Pooled StDev = Pooled standard deviation.

**Table 5:** Intestinal microbiota of Nile tilapia fed ALE–based diets.

Parameter (× 10^6^ CFU/g)^1^	ALE concentrations (g/kg)^2^				Pooled StDev^3^	*p* values	
	0.0	2.0	4.0	6.0		Linear	Quadratic
Total viable count	34.8^a^	22.3^b^	14.2^c^	12.2^c^	0.495	≤ 0.001	< 0.001

*Pathogenic bacteria*							
*Vibrio sp.*	24.2^a^	20.9^b^	7.7^c^	8.7^c^	0.631	≤ 0.001	≤ 0.001
*Flavobacterium sp.*	3.7^a^	3.5^a^	2.3^c^	2.8^b^	0.148	≤ 0.002	≤ 0.005
*Proteus sp.*	1.6^a^	1.3^ab^	1.1^b^	1.1^b^	0.122	≤ 0.001	≤ 0.001
*Pseudomonas sp.*	52.0^a^	2.2^b^	1.3^c^	1.4^c^	0.092	≤ 0.001	≤ 0.002

*Opportunistic bacteria*							
*Shigella sp.*	5.7^a^	3.2^b^	2.4^bc^	2.1^c^	0.250	≤ 0.001	≤ 0.001
*Escherichia coli*	16.8^a^	7.0^b^	3.2^c^	2.1^d^	0.228	≤ 0.001	≤ 0.001
*Bacillus sp.*	16.1^a^	10.5^b^	9.6^c^	8.9^d^	0.143	≤ 0.001	≤ 0.001
*Enterobacteriaceae sp.*	5.0^a^	6.1^ab^	5.4^ab^	4.0^b^	0.399	≤ 0.001	≤ 0.003
*Salmonella sp.*	16.6^a^	11.1^b^	9.5^b^	7.8^c^	0.342	≤ 0.001	≤ 0.001

*Note:* Means having the same superscript letter in the same row are not significantly different at *p* < 0.05.

^1^ALE = Atama leaves extract.

^2^Pooled StDev = Pooled standard deviation.

^3^CFU = Colony-forming unit.

**Table 6:** Hematology profiles of Nile tilapia fed ALE–based diets.

Parameters	ALE concentrations (g/kg)^1^				Pooled StDev^2^	*p* values	
	0.0	2.0	4.0	6.0		Linear	Quadratic
Packed cell volumes (%)	16.7^c^	17.9^c^	21.7^b^	24.5^a^	0.610	≤ 0.001	≤ 0.001
Hemoglobin (g/dL)	48.6^c^	57.0^b^	69.1^a^	71.7^a^	0.984	≤ 0.001	≤ 0.001
Red blood cells (× 10^6^/μL)	1.3^b^	1.3^ab^	1.4^b^	1.4^a^	0.036	≤ 0.001	≤ 0.003
White blood cells (× 10^3^/μL)	129.0^c^	154.2^b^	218.7^a^	221.2^a^	1.732	≤ 0.001	≤ 0.001
Platelets (× 10^6^/μL)	123.9^d^	183.3^c^	230.6^b^	242.9^a^	2.673	≤ 0.001	≤ 0.001
Lymphocytes (%)	46.5^d^	52.5^c^	61.5^b^	67.6^a^	0.707	≤ 0.001	≤ 0.004
Heterocytes (%)	45.5^a^	40.0^b^	33.2^c^	27.5^d^	2.131	≤ 0.001	≤ 0.001
Monocytes (%)	3.5^a^	3.4^a^	2.5^b^	2.5^b^	0.071	≤ 0.001	≤ 0.001
Eosinophils (%)	3.8^a^	3.5^b^	2.2^c^	2.2^c^	0.050	≤ 0.001	≤ 0.001
Basophils (%)	0.7^a^	0.6^b^	0.6^c^	0.2^d^	0.001	≤ 0.001	≤ 0.001

*Note:* Means having the same superscript letter in the same row are not significantly different at *p* < 0.05.

^1^ALE = Atama leaves extract.

^2^Pooled StDev = Pooled standard deviation.

## Data Availability

The data that support the findings of this study are available from the corresponding author upon reasonable request.

## Nomenclature

ALE: Atama leaves extract (ALE)
ALP: Alkaline phosphatase
ALT: Alanine aminotransferase
ANOVA: One-way analysis of variance
AST: Aspartate aminotransferase
Bas: Basophils (Bas, %)
CFU: Colony-forming unit
Creat: Creatinine
DM: Dry matter
DMSO: Dimethyl sulfoxide
DO: Dissolved oxygen
EMB: Eosin methylene blue
Eos: Eosinophils
FCR: feed conversion ratio
GC–MS: Gas chromatography–mass spectrophotometer
Hb: Hemoglobin
Het: Heterocytes
Lym.: Lymphocytes
MIC: Minimum inhibitory concentration
Mon: Monocytes
PCV: Packed cell volume
pH: Hydrogen ion concentration
PLT.: Platelets
RBC: Red blood cells
RT: Retention time
SGR: Specific growth rate
SR: Survival rate
SSA: *Salmonella Shigella* Agar
TSA: Tryptic soy agar
TSI: Triple sugar iron agar
VRBGA: Violet red bile glucose agar
WBC: White blood cells
WG: Weight gain
ZI: Zone of inhibition
